# How to Minimize the Attack Rate during Multiple Influenza Outbreaks in a Heterogeneous Population

**DOI:** 10.1371/journal.pone.0036573

**Published:** 2012-06-11

**Authors:** Isaac Chun-Hai Fung, Rustom Antia, Andreas Handel

**Affiliations:** 1 Department of Epidemiology and Biostatistics, College of Public Health, University of Georgia, Athens, Georgia, United States of America; 2 Department of Biology, Emory University, Atlanta, Georgia, United States of America; The Australian National University, Australia

## Abstract

**Background:**

If repeated interventions against multiple outbreaks are not feasible, there is an optimal level of control during the first outbreak. Any control measures above that optimal level will lead to an outcome that may be as sub-optimal as that achieved by an intervention that is too weak. We studied this scenario in more detail.

**Method:**

An age-stratified ordinary-differential-equation model was constructed to study infectious disease outbreaks and control in a population made up of two groups, adults and children. The model was parameterized using influenza as an example. This model was used to simulate two consecutive outbreaks of the same infectious disease, with an intervention applied only during the first outbreak, and to study how cumulative attack rates were influenced by population composition, strength of inter-group transmission, and different ways of triggering and implementing the interventions. We assumed that recovered individuals are fully immune and the intervention does not confer immunity.

**Results/Conclusion:**

The optimal intervention depended on coupling between the two population sub-groups, the length, strength and timing of the intervention, and the population composition. Population heterogeneity affected intervention strategies only for very low cross-transmission between groups. At more realistic values, coupling between the groups led to synchronization of outbreaks and therefore intervention strategies that were optimal in reducing the attack rates for each subgroup and the population overall coincided. For a sustained intervention of low efficacy, early intervention was found to be best, while at high efficacies, a delayed start was better. For short interventions, a delayed start was always advantageous, independent of the intervention efficacy. For most scenarios, starting the intervention after a certain cumulative proportion of children were infected seemed more robust in achieving close to optimal outcomes compared to a strategy that used a specified duration after an outbreak’s beginning as the trigger.

## Introduction

While vaccines have strongly reduced the morbidity and mortality burden for many infectious diseases that transmit from person to person, outbreaks of varying size and severity are still common, both in developed and developing countries. For these diseases, control measures such as drug therapy and prophylaxis, and social distancing (e.g. quarantine, school or airport closures) are useful strategies. It seems intuitive that the more stringent the control measure, the better the outcome, i.e. fewer infected people. This is true for a closed system, i.e. a scenario where a community experiences only a single outbreak. However, if multiple outbreaks of the same pathogen are possible and control might only be feasible for a limited duration (due to cost or other constraints), one can find the – somewhat counterintuitive – situation that too much control can be as bad as too little control [1].

**Figure 1:**
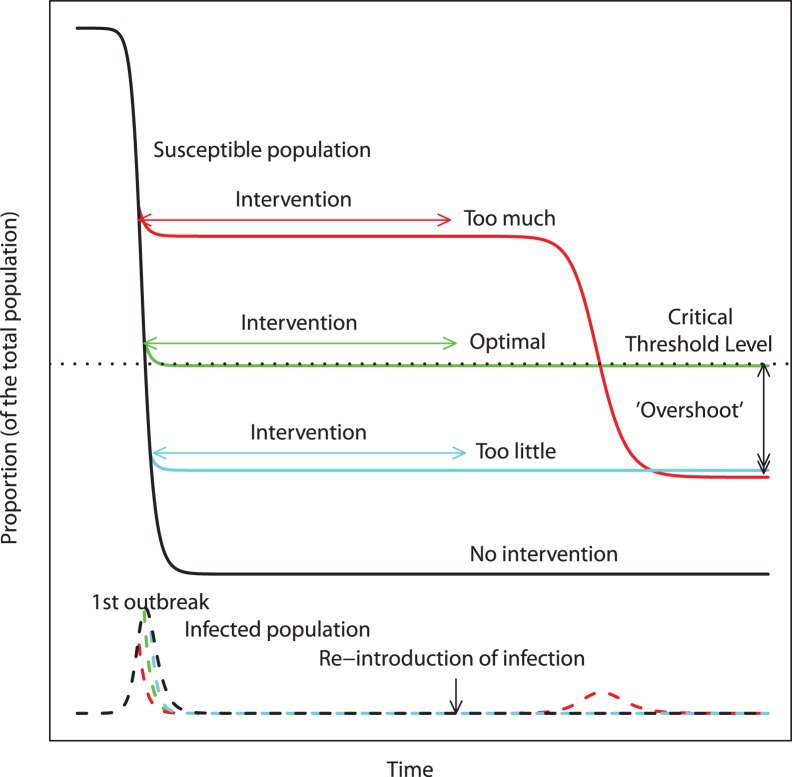
Illustration of the concept of optimal control for multiple outbreaks. We assume that multiple outbreaks can occur, with the intervention only being feasible during the first outbreak. If the intervention is weak (or absent), the first outbreak will be large enough to deplete the number of susceptible people below a critical threshold level (the herd immunity level below which effective reproduction number <1), such that if the infection is re-introduced, its effective reproductive number would be too low to cause a second outbreak (black and cyan lines). If the intervention is very strong, it is possible that after the first outbreak, the number of susceptible people remaining is large enough to support a second (uncontrolled) outbreak upon re-introduction of the pathogen, leading to an overall number of people infected that might be the same as that reached during just one outbreak (red line). In both the “too much” and “too little” intervention scenarios, the number of susceptible people drops below the critical threshold level, which defines the level of herd immunity. The excess drop is termed ‘overshoot’. The optimal intervention is one that minimizes the overshoot by allowing the susceptible population to drop to the critical threshold level during the first outbreak, such that a second outbreak cannot occur (green line). The solid lines represent the susceptible people and the broken lines represent the infected people.

This finding can be explained as follows: consider two outbreaks of the same pathogen. During the first outbreak, a public health intervention is implemented, which stops the outbreak (i.e. brings the effective reproduction number <1). However, the intervention does not provide immunity and people remain susceptible to the infection. At the end of the intervention, a few infected people are re-introduced into the population. If further interventions are not available, it will lead to an unmitigated second outbreak *if the number of susceptible people remaining after the first outbreak is high enough to have an effective reproduction number >1*. In other words, if the first outbreak has not depleted enough of the susceptible people for the population to reach a *critical threshold level* (the level at which enough herd immunity is achieved [2], [3]), a second outbreak will occur. As Figure 1 illustrates, this can lead to a situation where a control strategy that is too strong performs as poorly as an intervention strategy that is rather weak. The optimal strategy is one that brings the number of susceptible people down to the critical threshold level at the end of the first outbreak, thereby preventing a second outbreak. The excess number of infected people beyond those needed to reach the critical threshold level has been termed ‘overshoot’ [1]; an optimal intervention minimizes this overshoot and thereby minimizes the attack rate over all outbreaks. We have previously studied this scenario and it has also been recognized in the context of antiviral treatment of a drug sensitive influenza outbreak, followed by a drug resistant outbreak [1], [4]–[13].

In our previous work [1], we illustrated the multiple outbreak scenario using a simple mathematical Susceptible-Infected-Recovered (SIR) model of a homogeneous population for a generic infectious disease and unspecified, simple control. Here, we extend the model and analysis. For the current study, we decided to use a specific pathogen as an example. We chose influenza, since it is a pathogen for which such a multiple-wave situation has been observed [4], [5]. For instance, school closure for some period of time after the beginning of the first outbreak might prevent further infections, but as long as the pathogen still circulates in the population, re-opening the school will likely lead to another outbreak if enough students remain susceptible [14].

Instead of trying to build a realistic model for influenza transmission [15]–[24], we aimed for simplicity. We studied various control interventions in a heterogeneous population consisting of adults and children. This allowed us to investigate how variations in aspects such as composition and coupling between the two subpopulations, and strength, timing and other details of the control intervention affect the outcome. Our focus was on minimizing the cumulative attack rate.

We found that the optimal time to trigger an intervention depended on coupling between the two population sub-groups, the length of the intervention, the type of trigger and the population composition. Even a small degree of inter-group transmission led to coupling of both population groups (with different reproduction numbers) that was strong enough to synchronize the epidemic dynamics in both groups. This meant that the optimal time to trigger an intervention for both groups would be the same (instead of two different optimal times), and that with enough mixing between population sub-groups, homogeneous mixing models could be used to elucidate optimal control conditions. Short interventions shared similar optimal trigger conditions regardless of their efficacy. For long interventions to achieve optimal outcomes, stronger interventions should start late at the epidemic peak while weak interventions may start early. Using the cumulative proportion of children infected as the trigger provides more flexibility for decision-makers to obtain the optimal outcomes than using the time since the outbreak commences as the trigger. Overall, despite its simplicity, our model provided us with insights that will provide better information of epidemics and their interventions in real life.

## Materials and Methods

### The Model

**Figure 2:**
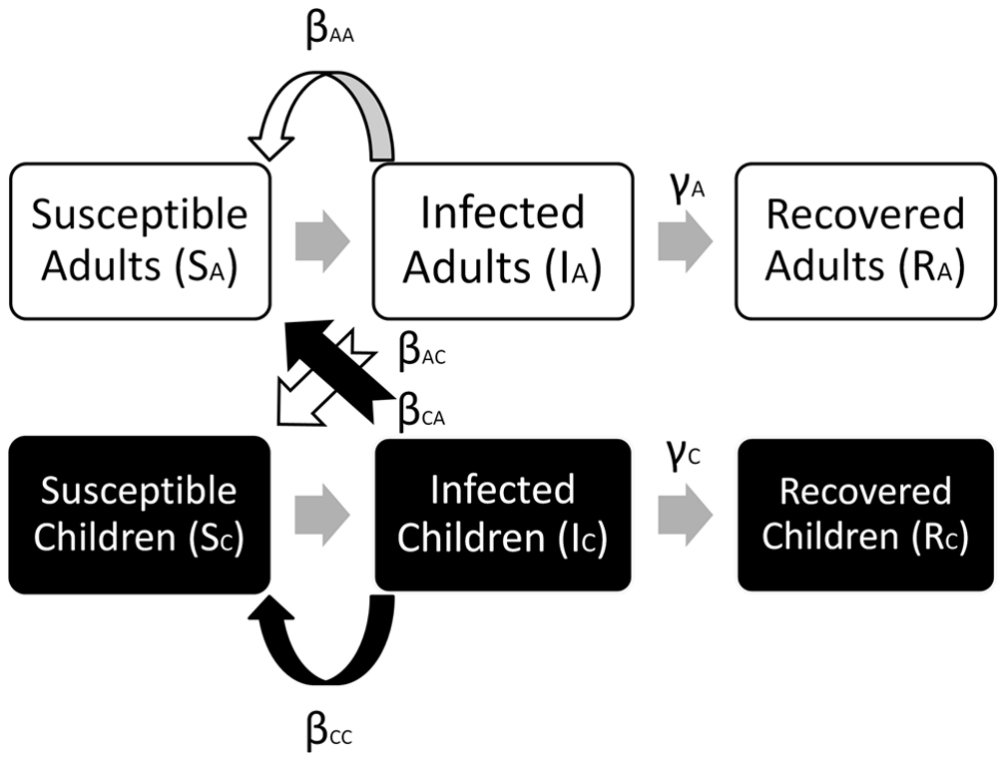
Flowchart illustrating the model. White boxes represent adults, while black boxes represent children. Light grey arrows indicate movements from one stage to another (susceptible to infected to recovered). White arrows represent infection of adults and children by contact with infected adults; likewise, black arrows represent a similar process with infected children.

We used a Susceptible-Infected-Recovered (SIR) model [2], [3], stratified by age into two categories: adults and children. Before the first outbreak, the whole population was assumed to be susceptible, an assumption that applied to a novel, pandemic influenza strain. Susceptible adults, *S_A_*, or children, *S_C_*, were infected by infected hosts of their own group (adults, *I_A_*; children, *I_C_*), at rates *β_AA_* and *β_CC_*, respectively. All infected hosts were assumed to be infectious. Infected adults infected children at the rate of *β_AC_* and infected children infected adults at the rate of *β_CA_*. An intervention reduced the rate of infection by a fraction (*f_AA_*, *f_AC_*, *f_CA_*, *f_CC_*). Infected adults and children recovered at rates of *γ_A_* and *γ_C_* respectively, and recovered adults *R_A_* and children *R_C_* were assumed to become immune to the infection. We normalized the whole population, *S_W0_* = *S_A0_*+ *S_C0_*, such that *S_W0_* = 1. Given that the duration of an influenza epidemic was short compared to human life expectancy, we did not include births, deaths and the aging process in our model. There was also no migration into or out of the population, apart from the re-introduction of infected people after the termination of the intervention to trigger the second outbreak. Figure 2 is a flowchart that illustrates the model. The model variables and parameters are summarized in Tables 1 and 2, the model equations are given by:
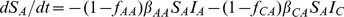
(1)

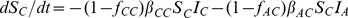
(2)

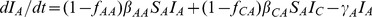
(3)

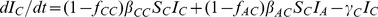
(4)

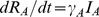
(5)

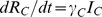
(6)


**Table 1:** Model variables.

Variables	Meaning	Initial value or definition	Comments or references
***S_A_***	Susceptible adults	(0.1 or 0.3 or 0.5 or 0.7 or 0.9) – *I_A_*	Chosen for illustrative purpose
***S_C_***	Susceptible children	1– *S_A_* – *I_C_*	Total population size is normalized to 1
***I_A_***	Infected adults	1e−6	Assuming 1 infected adult and 1 infected child in a city of 100,000.
***I_C_***	Infected children	1e−6	Ditto
***R_A_***	Recovered adults (who are immune to reinfection)	0	Assuming the population is totally susceptible at the beginning of the first outbreak
***R_C_***	Recovered children (who are immune to reinfection)	0	Ditto

**Table 2:** Model parameters.

Parameters	Meaning	Initial value or definition	Comments or references
***R_0A_***	Basic reproduction number (adults)	1.25 (default)	cf. Various estimates of R_0_ of 2009 pandemic influenza A (H1N1). See section “The model”.
***R_0C_***	Basic reproduction number (children)	2	Ditto.
***β_AA_***	Transmission coefficient from adults to adults	*R_0A_γ_A_*/(*S_A0_*+ *kS_C0_*)	Based on the definition of *R_0A._* See section “The model”.
***β_CC_***	Transmission coefficient from children to children	*R_0C_γ_C_*/(*S_C0_*+ *kS_A0_*)	Based on the definition of *R_0C._* See section “The model”.
***β_AC_***	Transmission coefficient from adults to children	k * *β_AA_*	See section “The model”.
***β_CA_***	Transmission coefficient from children to adults	k * *β_CC_*	See section “The model”.
***f_AA_***	Intervention efficacy to reduce transmission from adults to adults	0–1	Varied depending on the scenarios.
***f_AC_***	Intervention efficacy to reduce transmission from adults to children	0–1	Ditto.
***f_CA_***	Intervention efficacy to reduce transmission from children to adults	0–1	Ditto.
***f_CC_***	Intervention efficacy to reduce transmission from children tochildren	0–1	Ditto.
***γ_A_***	Recovery rate of adults	1/4.8 day^−1^	[47]. It is the reciprocal of viral shedding period of an infected adult.
***γ_C_***	Recovery rate of children	1/8 day^−1^	[48]. It is the reciprocal of viral shedding period of an infected child.

Unless otherwise specified, the parameter values and initial conditions were chosen as given in Tables 1 and 2. We investigated the impact on the results for changes in the different parameters and initial conditions and reported those that were influential. Those changes with little influence (e.g. in the fraction of initially infected people) were not reported.

Simulations of the mathematical model were implemented in R 2.12.2 (http://cran.r-project.org/). The code is available upon request.

The basic reproduction number, *R_0_*, is a measure of the transmissibility of an infection, and is defined as the number of secondary cases caused by one infectious individual being introduced into a totally susceptible population. Here, with adults and children as two sub-groups of the population, the basic reproduction number of the adults (*R_0A_*) refers to the number of secondary cases (adults and children) of infection caused by an infectious adult introduced into a totally susceptible population in the absence of any intervention; and the basic reproduction number of the children (*R_0C_*) is similarly defined. Mathematically, they are given by:
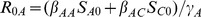
(7)

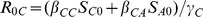
(8)where *S_A0_* and *S_C0_* refer to the initial proportions of susceptible adults and children in the population. We assumed that *R_0A_* < *R_0C_*. The values used in most parts of this paper (*R_0A_* = 1.25 and *R_0C_* = 2) are in line with current findings of the influenza A (H1N1) 2009 pandemic (cf. various published estimates of *R_0_* (or the effective reproduction number, *R*) in different populations [25]–[33]).

We could use the values for R_0A_ and R_0C_ to compute the transmission terms, *β.* However, not enough information was available to determine all 4 transmission terms. Therefore, we made the simplifying assumption that the ratio of cross-transmission between groups (i.e. from adults to children and from children to adults) and within-group transmission was the same for adults and children, and therefore, *β_AC_* = k * *β_AA_* and *β_CA_* = k * *β_CC_*. In other words, we assumed that the proportion of the within- and between-group mixing of adults and children was equal. While no model captures every aspect of the bio-social reality of influenza transmission, we believe that to have fixed *R_0C_* and *R_0A_* in the model reflects the infectiousness (a biological property) of the virus in a hypothetical “ideal” situation where its transmission takes place in a totally susceptible population. As the demographic composition of a population changes, it affects the virus’ transmission, and therefore changes the four βs. The remaining transmission terms were then given by,
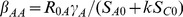
(9)

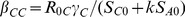
(10)


### ‘Time trigger’ and ‘Population Trigger’

After an infectious disease outbreak occurs, there is some delay until interventions are implemented. This delay can either be due to logistical constraints, i.e. it takes time to ramp up a control effort, or due to other considerations, i.e. it may only make sense to close a school once enough students are infected and it is clear that an outbreak is occurring. We investigated both situations. For the first scenario, which we called ‘Time trigger’, the intervention was started on a given day after the beginning of the first outbreak. For the second scenario, which we called ‘Population trigger’, the intervention was started once the cumulative fraction of people in the population who got infected reached a specific level.

### Cumulative Attack Rates

In this study, we quantified the effects of different intervention schemes by determining the cumulative attack rate at the end of all, i.e. both the first and the second, outbreaks. The cumulative attack rates were determined by recording the proportions of susceptible people at the end of the last outbreak and were defined as follows:

Cumulative attack rate, whole population:
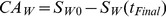
(11)


Cumulative attack rate, adults, whole population as the denominator:
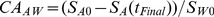
(12)


Cumulative attack rate, children, whole population as the denominator:
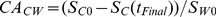
(13)


Cumulative attack rate, adults, initial adults’ proportion as the denominator:
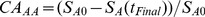
(14)


Cumulative attack rate, children, initial children’s proportion as the denominator:
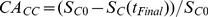
(15)


Note that since we normalized the total population, i.e. we chose S_W0_ = 1, we had the following simple relations between the different attack rates: CA_AA_ = CA_AW_/S_A0_, CA_CC_ = CA_CW_/S_C0_, and CA_W_ = CA_AW_ + CA_CW_.

## Results

### The Impact of Inter-group Transmission

#### No inter-group transmission

**Figure 3:**
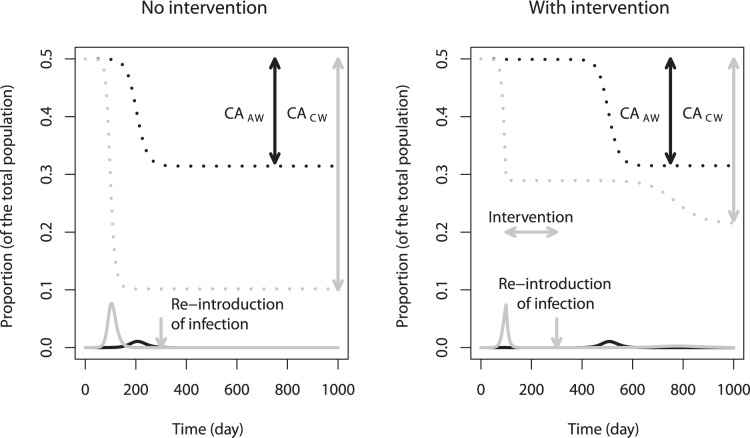
Time series of a simulated epidemic in a population in which there is no transmission between adults (black) and children (grey), in the absence (left) or presence (right) of an intervention. Broken line: susceptible; solid line: infected. CA_AW_: Cumulative attack rate (adults, with whole population as the denominator); CA_CW_: Cumulative attack rate (children, with whole population as the denominator). Infection was re-introduced into the population on day 300. The intervention (*f_AA_* = *f_AC_* = *f_CA_* = *f_CC_* = 1) started on day 100 (when the children’s epidemic is at its peak) and lasted until the first outbreak was over. β_AC_ = β_CA_ = 0; R_0A_ = 1.25; R_0C_ = 2. 50% adults; 50% children. All other parameters and initial conditions are listed in Tables 1 and 2.

We started with a population comprised of 50% adults and 50% children, with different reproduction numbers for adults and children that were both greater than 1 (*R_0A_* = 1.25 and *R_0C_* = 2). To obtain a basic understanding of the system, we first investigated a scenario for which there was no transmission between adults and children (*β_AC_* = *β_CA_* = 0). In other words, we had two outbreaks in two independent populations. As children had a higher reproduction number than adults, the epidemic spread faster and reached its peak earlier among children as compared to adults. In the absence of any intervention, the fraction of the susceptible population in both populations dropped below the critical threshold level, such that a second outbreak could not occur (Figure 3, left panel).

**Figure 4:**
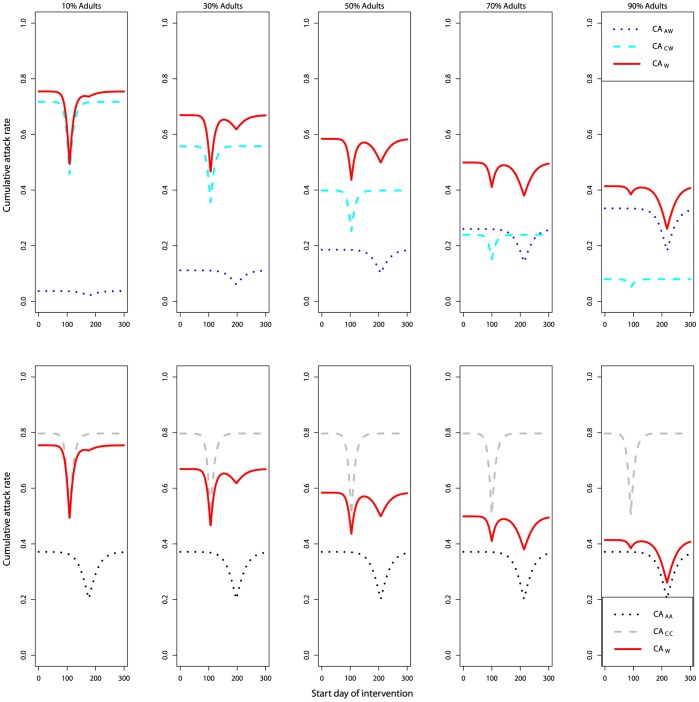
Cumulative attack rates (CA) against the start day of intervention, in the absence of inter-group transmission (β_AC_ = β_CA_ = 0). In the upper row, the denominator in the adults’ and children’s CA is the whole population (S_W0_ = 1); blue dotted line: CA_AW_; cyan broken line: CA_CW_; red solid line: CA_W_ = CA_AW_ + CA_CW_. In the lower row, the denominator in the adults’ and children’s CA is their respective proportion in the whole population (SA_0_ and SC_0_ respectively); black dotted line: CA_AA_; grey broken line: CA_CC_; red solid line: CA_W_ = S_0A_*CA_AA_ + S_0C_*CA_CC_. Proportion of adults in the population, from left to right: 10%, 30%, 50%, 70% and 90%. Intervention efficacy, *f_AA_* = *f_AC_* = *f_CA_* = *f_CC_* = 1. R_0A_ = 1.25, R_0C_ = 2; long intervention; interrupt all routes of transmission. All other parameters and initial conditions are listed in Tables 1 and 2. For the definition of the different cumulative attack rates, please refer to the Materials and Methods section, “Cumulative attack rates”, in the main text.

The situation differed once the intervention was introduced (Figure 3, right panel). For this example, we started a perfect intervention that interrupted all routes of transmission (*f_AA_* = *f_AC_* = *f_CA_* = *f_CC_* = 1) on day 100 after the beginning of the outbreak and continued to apply the intervention until the first outbreak was over. We then re-introduced a small number of infected people. Since the intervention was strong enough to keep the number of susceptible people in both populations above the critical threshold level (in fact, the adult population remained almost uninfected), a second outbreak – without any intervention measures applied – occurred. The cumulative attack rate at the end of the *second* outbreak was then recorded. The first question we wanted to address was: Is there an optimal time to start the intervention during the first outbreak? Intuitively – based on Figure 1– we would expect that too early/strong and too late/little were both less than optimal, therefore there should be some intermediate time at which starting the intervention was optimal. (Note again that so far we assumed that once an intervention was started, it would last for the duration of the outbreak. We relaxed this assumption later). As expected, we found that there was an intermediate time that was optimal in reducing the number of infections (Figure 4). Also not surprisingly, we observed that the best time to start the intervention for adults was different from that for children. For both populations, to achieve the optimal outcome, the intervention needed to start at the time of the peak of the epidemic curve when the proportion of the susceptible population remaining approached the critical threshold level (i.e. the effective reproductive number was 1). Because the two populations had different outbreak dynamics, their peaks, and therefore optimal time of intervention, differed. Since the cumulative attack rate for the whole population was the sum of the cumulative attack rates for the two populations, it had two minima, corresponding to the optimal intervention start times for adults and children, respectively. Obviously, the respective fraction of adults and children in the population influenced the importance of each minimum: If one group dominated, this became the dominant minimum (Figure 4 upper panels). Also we noted that, in the absence of coupling, the change in attack rate for each population using the respective proportions of adults and children as the denominators for their cumulative attack rates, i.e. CA_AA_ and CA_CC_, stayed the same even if the composition of the population changed (Figure 4 lower panels).

**Figure 5:**
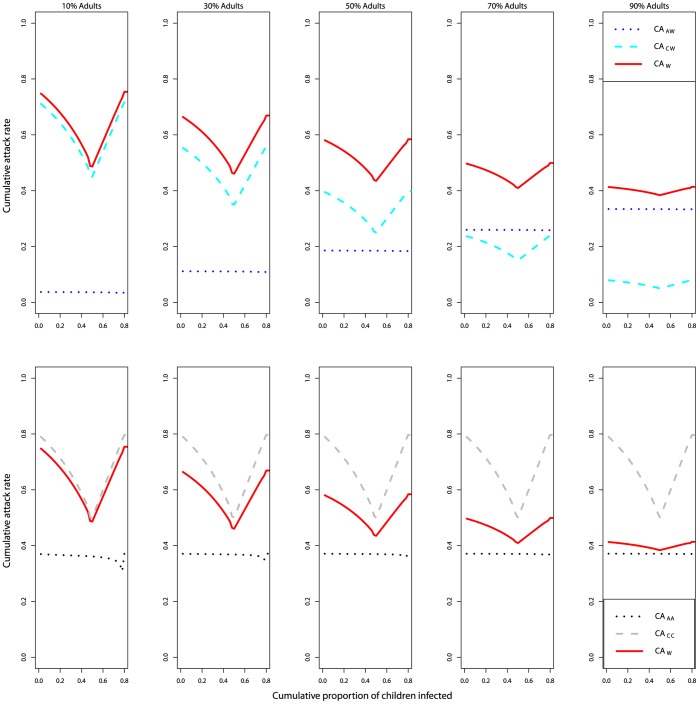
Cumulative attack rates (CA) against children’s population trigger (defined as the cumulative proportion of children infected), in the absence of inter-group transmission (β_AC_ = β_CA_ = 0). Everything else as described in Figure 4 legend. Note that the x-axis does not go beyond a threshold level of 0.8 since even an unmitigated outbreak does not reach a higher attack rate among the children and therefore an intervention would not be triggered.

**Figure 6:**
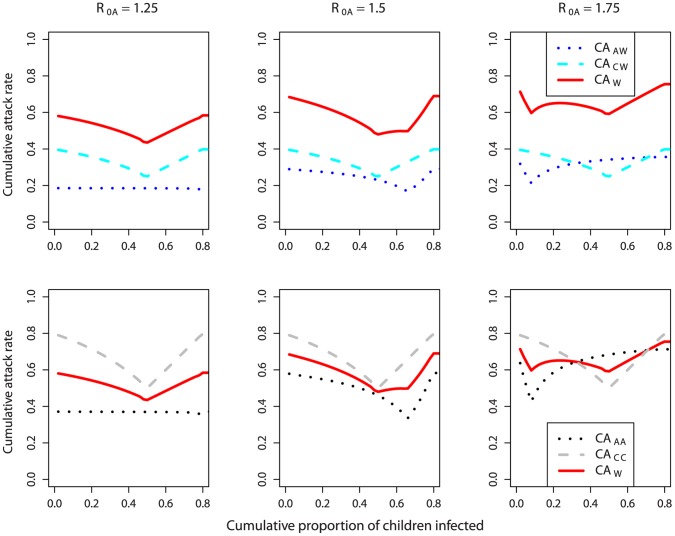
Cumulative attack rates against children’s population trigger, in the absence of inter-group transmission (β_AC_ = β_CA_ = 0). Values of R_0A_ are varied: R_0A_ = 1.25 (left); 1.5 (middle); 1.75 (right). For all panels, R_0C_ = 2; 50% adults; 50% children. Everything else as described in Figure 5 caption.

Next, we studied how the cumulative attack rates changed if instead of using a ‘time trigger’, i.e. starting an intervention after a certain period of time had lapsed since the outbreak started (either involuntarily, due to delays in the response, or planned to optimize overall outcome, as just described), the intervention was triggered based on the number of infected people (i.e. ‘population trigger’). It is likely that one might want to start an intervention only once the proportion of infected people reaches a certain level. While infections among adults, children, or both adults and children can serve as a trigger for interventions, in practice for an infection like influenza, children are likely the best trigger. It is because they are likely the first ones to be infected (larger *R_0_*) and because measuring the level of infection among them will be relatively easy, e.g. through surveillance in schools. We therefore focus here on children as the trigger for interventions (‘children’s population trigger’) and briefly discuss results for the total population as a trigger in Supporting Information, Appendix S1A. Using the fraction of infected children as an intervention trigger led to different results (compare Figures 4 and 5). Only one minimum for CA_W_ was observable, which coincided with the minimum for the children. While there was a minimum for the adult attack rates, it was very small and only visible in the bottom left panel. Therefore the overall attack rate followed that of the children. The reason why there was no significant reduction in the attack rate among the adults had to do with the differences in the basic reproduction number between the populations. For the values chosen here (*R_0A_ = *1.25, *R_0C_*_ = _2), the epidemic wave of the adults lagged far behind that of the children (cf. Figure 3). By the time the children’s cumulative attack rate reached the level at which the intervention was triggered, the adults’ epidemic curve was nowhere close to its peak. This meant that independent of trigger level, the intervention among the adults had a similar impact, namely preventing most adult infections during the first outbreak, followed by a large, uncontrolled, second outbreak, leading to an overall minimal reduction in adults’ cumulative attack rate. If *R_0A_* was increased such that the dynamics of the two outbreaks was closer, one observed the ‘two-minima’ phenomenon again (Figure 6). Note that in all figures displaying results of scenarios using ‘children’s population trigger’, the x-axis did not go beyond a threshold level of 0.8 since even an unmitigated outbreak did not reach a higher attack rate among the children and therefore an intervention would not be triggered.

#### Coupling through inter-group transmission

**Figure 7:**
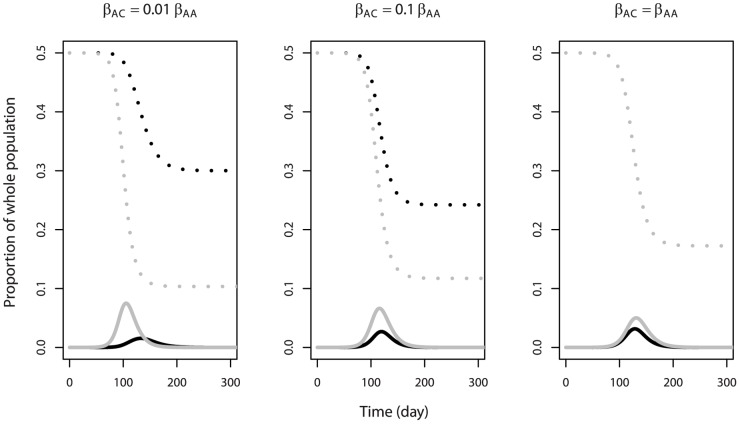
Time series of a simulated epidemic in a population in which transmission between adults (black) and children (gray) is 1% of intra-group transmission (β_AC_ = 0.01 * β_AA_; β_CA_ = 0.01 * β_CC_; left), or 10% (β_AC_ = 0.1 * β_AA_; β_CA_ = 0.1 * β_CC_; middle), or 100% (β_AC_ = β_AA_; β_CA_ = β_CC_; right), in the absence of intervention. Broken line: susceptible; solid line: infected. In the right panel, the black and gray broken lines overlap each other exactly. R_0A_ = 1.25; R_0C_ = 2. 50% adults; 50% children. All other parameters and initial conditions are listed in Tables 1 and 2. The reason why in the right panel, the curves of susceptible adults and children overlapped, while the curves of infected adults and children did not, was that adults and children had different rates of recovery and therefore their average durations in the model compartment of the infected/infectious were different.

**Figure 8:**
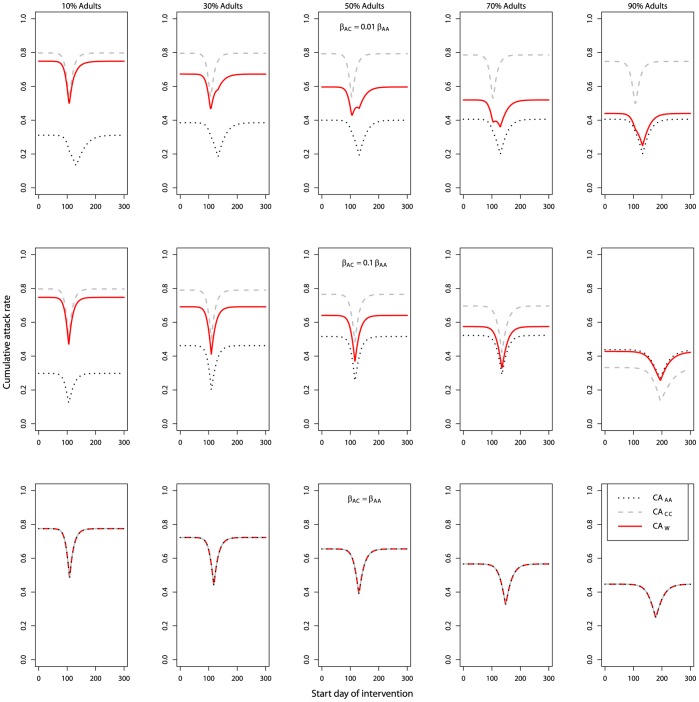
Cumulative attack rates against the start day of intervention in the presence of inter-group transmission. Transmission between adults and children was 1% of intra-group transmission (β_AC_ = 0.01 * β_AA_; β_CA_ = 0.01 * β_CC_; upper row), or 10% (β_AC_ = 0.1 * β_AA_; β_CA_ = 0.1 * β_CC_; middle row), or 100% (β_AC_ = β_AA_; β_CA_ = β_CC_; lower row). The fraction of adults in the population was (from left to right) 10%, 30%, 50%, 70%, and 90%. Black dotted line: adults (CA_AA_); grey broken line: children (CA_CC_); red solid line: whole population (CA_W_). Like the no-coupling scenario, the CA_W_ curve shifted lower if there were more adults and higher if there were more children. In the lower panels, all three lines overlap with each other. R_0A_ = 1.25, R_0C_ = 2; 50% adults; 50% children; long intervention; interrupt all routes of transmission; intervention efficacy, *f_AA_* = *f_AC_* = *f_CA_* = *f_CC_* = 1. All other parameters and initial conditions are listed in Tables 1 and 2.

**Figure 9:**
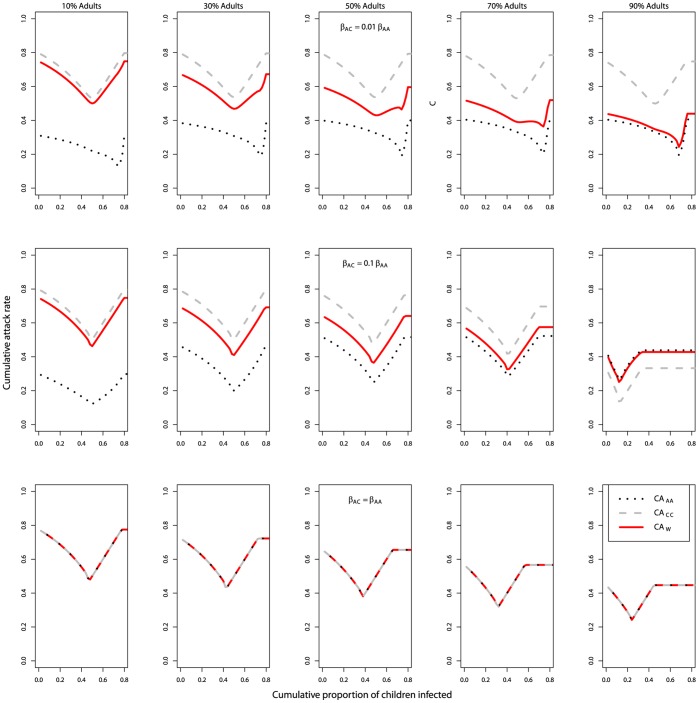
Cumulative attack rates against children’s population trigger in the presence of inter-group transmission. Everything else as described in Figure 8 caption. Note that again the x-axis does not go beyond a threshold level of 0.8 since even an unmitigated outbreak (even in the absence of coupling) did not reach a higher attack rate among the children and therefore intervention would not be triggered.

So far, we have considered two independent outbreaks among adults and children, with no inter-group transmission. This was done to gain some basic understanding of the system. However, for any realistic situation, some level of inter-group transmission generally occurs [34]–[36]. Coupling of the populations induced by such inter-group transmission can lead to synchronization between the dynamics in the different populations [3]. We were interested to see how optimal control strategies might change in the presence of coupling between adults and children. As one might expect, if coupling was very low, for example, inter-group transmission being 1% of intra-group transmission (*β_AC_* = 0.01 * *β_AA_*; *β_CA_* = 0.01 * *β_CC_*), the two outbreaks still showed differing dynamics (Figure 7, left panel). The two-minima phenomenon could still be observed in the CA_W_ curves if time was used as the intervention trigger (in the cases of 50% and 70% adults, upper panels in Figure 8), though not as pronounced as in the ‘no coupling’ scenario (cf. Figure 4). Interestingly, and in contrast to the results shown above, two minima were now observed in the CA_W_ curve (for the scenarios of 50% and 70% adults) if the children’s population trigger was used (compare Figure 5 with upper panels in Figure 9). This was because coupling led to an accelerated outbreak among the adults. This was similar to the case of an increase in *R_0A_* in the absence of inter-group transmission (cf. Figure 6), and had similar effects, i.e. giving two minima. If the population was dominated by one group or another, only one minimum was observed (as in the cases of 10%, 30% and 90% adults), as the other group was too small to have a noticeable impact on CA_W_.

If inter-group transmission was higher, e.g. 10% of intra-group transmission (*β_AC_* = 0.1 * *β_AA_*; *β_CA_* = 0.1 * *β_CC_*), we observed that the epidemic curves for adults and children became almost fully synchronized (middle panel in Figure 7). The optimal starting condition for the intervention for children and that for adults coincided and therefore there was only one minimum in the CA_W_ curve against either the time trigger (middle panels in Figure 8) or children’s population trigger (middle panels in Figure 9). As the proportion of adults increased from 10% to 90% (middle panels in Figure 8), the overall *R_0_* was lowered and the outbreak reached its peak later. Therefore, the optimal time to trigger the intervention shifted to a later time.

If the level of coupling was increased to the point where the rate of transmission from an infected adult to a susceptible adult was equal to that from an infected adult to a susceptible child (*β_AC_* = *β_AA_*), and likewise, that from an infected child to a susceptible child was equal to that from an infected child to a susceptible adult (*β_CA_* = *β_CC_*), it was found that for any given time trigger or population trigger, the CA_AA_, CA_CC_ and CA_W_ curves perfectly overlapped with one another for any given time trigger or population trigger, provided that the intervention interrupted all routes of transmission equally (lower panels in Figure 8 and Figure 9). This can be explained by the fact that for this situation, the force of infection was the same for both children and adult populations, independent of the proportions of adults and children within the population. Therefore, the dynamics of the susceptible people in both populations, and the final cumulative attack rates, were identical. This result can also be shown analytically (See Supporting Information, Appendix S1B).

In contrast to the no-coupling scenario, we found that in the presence of coupling, CA_AA_ and CA_CC_ varied as the proportions of adults and children in the population changed. CA_CC_ was lower if a large fraction of the population consisted of adults. This was due to the fact that for a fixed *R_0C_*, an increase in the proportion of adults in the population led to a higher fraction of adults and a smaller fraction of children being infected by an infectious child. The simultaneous increase of children being infected by infectious adults was lower since *R_0A_* was lower. Therefore, it led to an overall reduction in the outbreak among the children. The flipside of this argument also explained the increase in CA_AA_. Similarly, for the 10% adult scenarios in which most of the population were children, in the presence of coupling, more children and fewer adults were infected by an infectious person, leading to a lower CA_AA_. In the 90% adult scenario with a moderately low coupling (Figure 8, middle row, right panel), the relatively modest increase in CA_AA_ (as compared to the no-coupling scenario) was a result of CA_AW_ reaching its limit and a larger denominator (S_A0_) that led to a CA_AA_ that was smaller than that in the 30%, 50% and 70% scenarios.

### Different Types of Intervention

So far, we considered interventions that were 100% effective (*f* = 1), applied to each group, and that lasted for the duration of the first outbreak. These assumptions were used to study the simplest scenarios first, but were unrealistic. We then investigated how limiting the strength and duration of the intervention affected the cumulative attack rate. We also studied a scenario in which the intervention was applied to one group only (interrupting children-to-children transmission by school closure).

#### Strength of intervention

**Figure 10:**
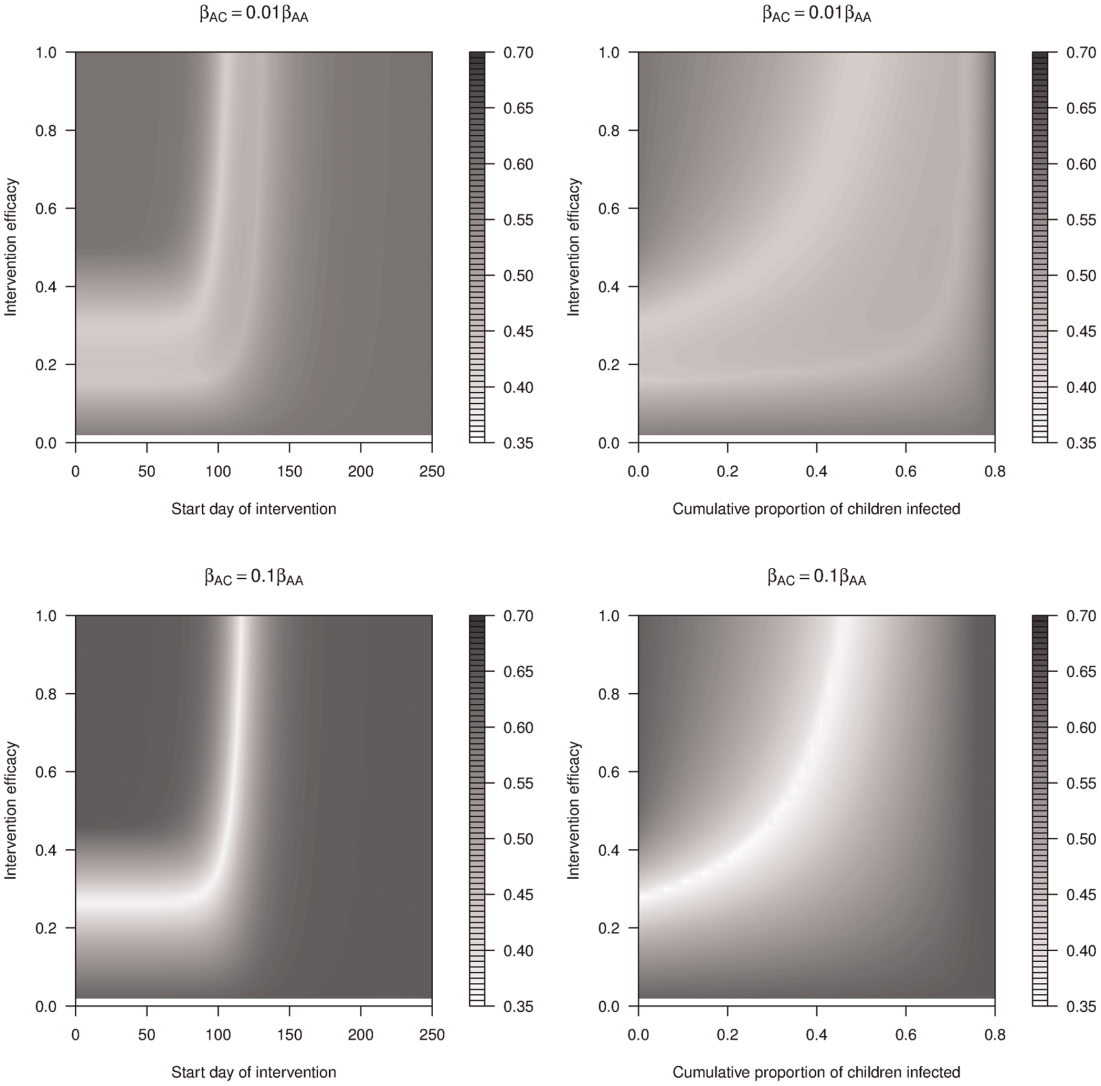
Cumulative attack rate of the whole population (CA_W_) under long intervention with different levels of efficacy against different time trigger (the start day of intervention, left column) and population trigger (cumulative proportion of children infected, right column). β_AC_ = 0.01 * β_AA_; β_CA_ = 0.01 * β_CC_ (upper row); β_AC_ = 0.1 * β_AA_; β_CA_ = 0.1 * β_CC_ (lower row). R_0A_ = 1.25, R_0C_ = 2; 50% adults; 50% children (similar patterns were observed in populations with different proportions of adults and children, not shown); long intervention; interrupt all routes of transmission. All other parameters and initial conditions are listed in Tables 1 and 2. The data ranged from intervention efficacy of 0.02 to 1 with intervals of 0.02. This explains why there was no colour in the contour plots from intervention efficacy 0 to 0.02. Note that beyond a threshold level of 0.8, the intervention was not triggered, as CA_AA_ never reached 0.8 even in the absence of any intervention.

We began the analysis of the impact of different types of interventions by varying intervention strength. Figure 10 shows how interventions of varying efficacy influenced the time or infected population proportion at which the intervention should be triggered to minimize the cumulative attack rate (whole population, CA_W_). Here we only show the scenarios in which inter-group transmission was present and smaller than intra-group transmission (β_AC_ = 0.01 * β_AA_; β_CA_ = 0.01 * β_CC_; and β_AC_ = 0.1 * β_AA_; β_CA_ = 0.1 * β_CC_), as we considered the other cases analyzed above (β_AC_ = β_CA_ = 0; or β_AC_ = β_AA_; β_CA_ = β_CC_) less realistic and nothing qualitatively new was found in those scenarios.

We produced contour plots where we varied the triggers of the intervention as in the figures above, and now additionally varied the intervention efficacy. For 100% intervention efficacy (f = 1), the results from section 3.1 were again observed: there were two minima when coupling was low (β_AC_ = 0.01 * β_AA_; β_CA_ = 0.01 * β_CC_, Figure 10 upper row) and there was only one minimum when coupling increased (β_AC_ = 0.1 * β_AA_; β_CA_ = 0.1 * β_CC_, Figure 10 lower row). As the intervention efficacy decreased, it was found that in both cases, the minima persisted. Over a large range of intervention efficacies, there was a combination of strength and timing that produced close to optimal results with respect to reduction in the attack rate. The minima followed a J-shape: If an effective intervention was available, it was best to start it close to the epidemic peak(s); for a less effective intervention, an earlier start was the best. This was not surprising, as very low efficacy meant that the intervention was so weak that the first outbreak led to a drop in the number of susceptible people below the critical threshold level, no matter how early or late the intervention started. In such a case, the earlier was always better.

Another interesting feature was that the two minima for the low coupling case remained as efficacy was varied. The double J-shape implied that while for high intervention efficacies, there were two minima with respect to intervention triggers, for low intervention efficacies, the reverse also applied: For a given intervention triggers, there could be two levels of intervention strength that produced similar results, with less optimal levels between them. For example when coupling was low (β_AC_ = 0.01 * β_AA_; β_CA_ = 0.01 * β_CC_; Figure 10, top left panel), when the intervention started at day 0, an intervention of efficacy f ≈ 0.3 could achieve the optimal outcome. However, if an available intervention only had efficacy f ≈ 0.2, it would be advisable to implement it less then fully such that one ended up with f ≈ 0.15 and a better reduction in attack rate.

It was also observed in Figure 10 that the gradient of CA_W_ was relatively steep in the contour plots against the time trigger (left column) as compared with that against the population trigger (right column). This implies that using a specified level of infections as intervention trigger seems more robust in achieving optimal control outcomes, especially given that the exact date of an outbreak’s beginning is usually more difficult to ascertain than the number of infected people (e.g. by serosurveillance).

#### Length of intervention

**Figure 11:**
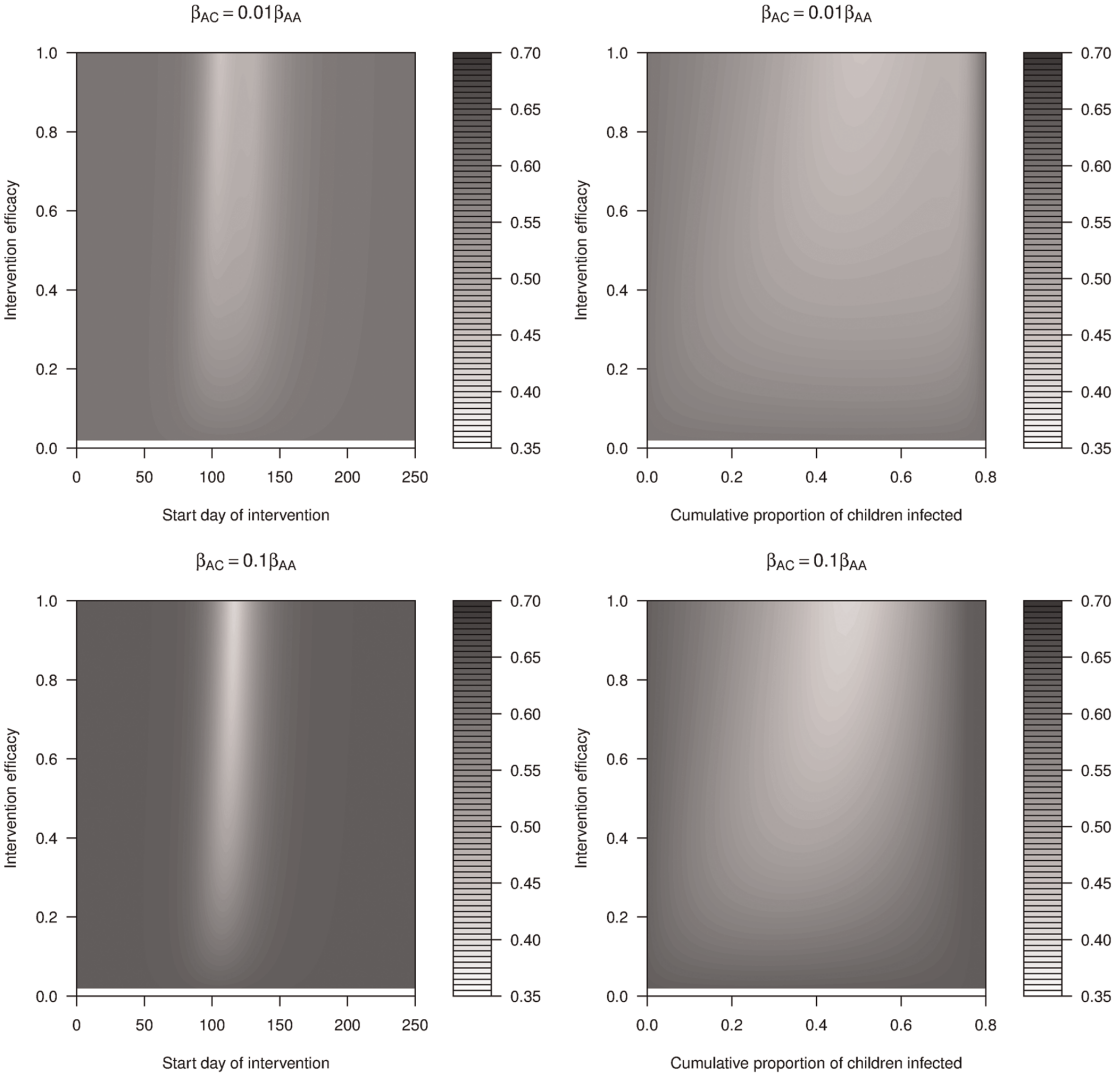
Cumulative attack rate of the whole population (CA_W_) under 28-days intervention with different levels of efficacy against different time triggers (start days of intervention, left) and population triggers (cumulative proportion of children infected, right). The legend colour panel displays CA_W_. β_AC_ = 0.01 * β_AA_; β_CA_ = 0.01 * β_CC_ (upper row); β_AC_ = 0.1 * β_AA_; β_CA_ = 0.1 * β_CC_ (lower row). All other details are the same as Figure 10. Compared to Figure 10, it is notable that the J-shaped contours of Figure 10 are replaced by vertical minima in Figure 11. In other words, weak interventions that started early would not lead to the optimal outcome if its duration was short.

For all the previous results, the intervention lasted until the first outbreak was over. For comparison, we simulated **short intervention** scenarios where the intervention was restricted to a length of 28 days (arbitrarily chosen for the purpose of illustration). Several differences between this short and the previous long interventions were notable (compare Figures 10 and 11). In the above sections, we found that a weak intervention, if started early and maintained long enough, could help mitigate the outbreak enough that the number of susceptible people would not drop much below the critical threshold level during the first outbreak. For a short intervention, this did not happen. If it started early, it would only slow the initial stages of the outbreak, but once the intervention was removed, the outbreak would continue in full force. Therefore, the J-shape observed previously was not seen for short interventions.

Instead, the best time to start a short intervention was at or somewhat before the peak of the outbreak, where it would have its strongest impact. As intervention efficacy decreased, the maximum reduction in attack rate that was achievable also decreased, the time of optimal intervention changed little.

As for the long intervention, one found again that using a specified level of infections as intervention trigger seems more robust towards making small errors in starting the intervention as compared to a time trigger.

#### School closure

**Figure 12:**
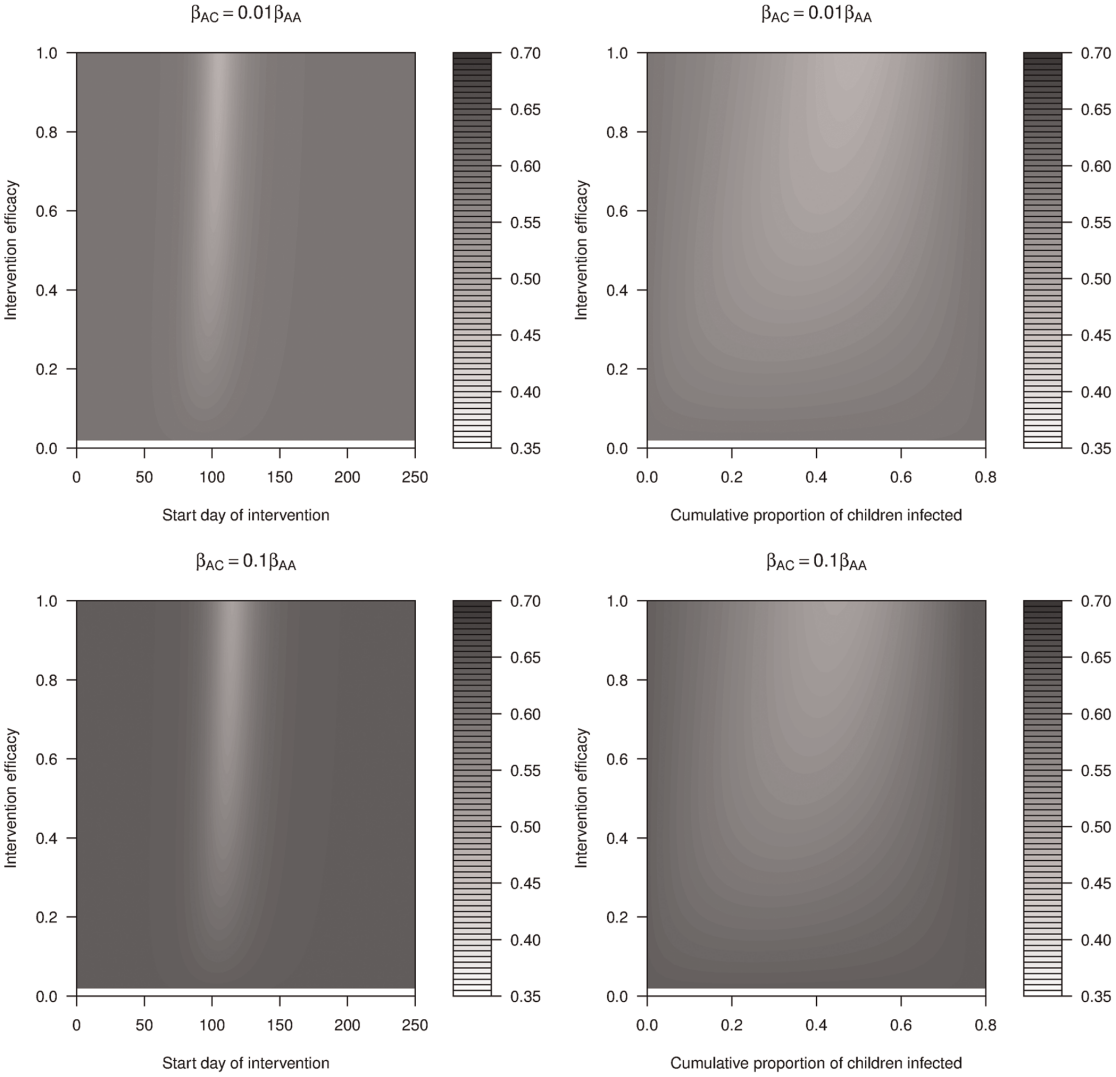
Cumulative attack rate of the whole population (CA_W_) under school closure with short intervention (28 days) with different levels of efficacy against time trigger (different start day of intervention, left) and population trigger (cumulative proportion of children infected, right). The legend colour panel displays CAW levels. All other parameters follow Figure 11.

In all the previous simulations, the intervention interrupted all routes of transmission to an equal extent (comprehensive intervention). For comparison, ‘school closure’ scenarios were simulated, in which only children-to-children transmission was interrupted (for 28 days) by the intervention (f_AA_ = f_AC_ = f_CA_ = 0; f_CC_ >0). For such a situation, intervention mainly reduced the outbreak among the children and had little impact on the outbreak among the adults. Reduction in overall attack rate was dominated by reduction in the children’s cumulative attack rate. Therefore, only a single minimum, that of the children, was observed in the total attack rate (compare Figures 11 and 12 top left). The same absence of two minima was seen if school closure was assumed to be long, i.e. lasting the duration of the outbreak (not shown). As expected, if only transmission between children was reduced, efficacy had to be higher to achieve a similar optimal outcome compared to a reduction of all transmission routes. Otherwise, the overall qualitative features found and discussed above for comprehensive intervention applied to this case where intervention was applied only to a certain route of transmission.

## Discussion

Using influenza as an example, we investigated how a single intervention strategy should be optimally implemented to reduce the overall attack rate in a heterogeneous population experiencing two outbreaks of the same infection. The specific scenario under study was that an intervention that did not provide immunity was applied to the first, but not the second outbreak. We used the cumulative attack rate over both outbreaks - i.e. the final size [37] of the epidemic over two outbreaks - as a measure of the success of the intervention.

Our simulations show that even low levels of inter-group transmission in a heterogeneous population can lead to temporal synchronisation of epidemic peaks and therefore the optimal time to start the intervention with regard to the two population sub-groups. It seems that there is a fair amount of inter-group transmission in different human populations [34]–[36]. Thus it is reasonable to conclude that with regard to the conditions of the commencement of intervention in the settings defined above (time trigger or population trigger), adults and children can be treated as a single homogeneous population even if the basic reproduction numbers among them are different. Our findings assure us that even simple models that assume a homogeneous population, e.g. [1], can shed light upon some of the essence of an infectious disease epidemic.

We found that the proportions of adults and children in the population affected the timing of an optimal intervention. Given the basic reproduction number among children was higher than that among adults (assuming both R_C0_ and R_A0_ were fixed), as the proportion of adults in the population increased, the peak of the epidemic shifted later and the children’s cumulative attack rate attained by then was lower. Therefore, the best time to commence the intervention in our specific scenario would shift later, and the optimal population trigger (the proportion of children infected among all children) would be lower. The overall population infected (CA_W_) would be lower as well. Henceforth the composition of a mixed population of sub-groups with different basic reproduction numbers affects the optimal condition to start an intervention and the cumulative attack rate thereby achieved.

Our results shed light on the relationship between the strength of an intervention, its length, its trigger condition and the coupling of the population sub-groups. While population mixing is usually an intrinsic feature in a particular population, the other three are attributes of an intervention programme and can often be manipulated to achieve the optimal outcome. We found that there was usually more than one combination of these attributes that would generate the optimal outcome for an intervention. This suggests that for a given outbreak, if parameters such as R_0_ and the efficacy of the intervention can be estimated, one can determine the right timing for the intervention to minimize attack rate over multiple outbreaks, in resource-constrained settings. Our study suggests that using the fraction of infected people in a sentinel group (e.g. schoolchildren) is a more robust trigger for interventions and is preferable to trying to start an intervention a given number of days after the beginning of the outbreak, which is often poorly known. Nonetheless, we acknowledge the difficulty in surveying serosurveillance in real time, especially as the number of infected persons increases rapidly around the optimal time at which the intervention should start.

While it was true that for weak interventions of long duration, the earlier it started, the better was the outcome, it did not seem to be applicable to the more realistic situation of an intervention of limited duration, independent of efficacy. This suggests that interventions that can be maintained (and of which the efficacies are often low, e.g. encouraging people to wash their hands more often for the duration of an outbreak) should be advocated early. Interventions that are costly to maintain (but are often of high efficacies, e.g. school or airport closure, the use of facemasks in non-clinical settings) should be implemented later during the outbreak.

For most of our analysis, we assumed control that acted equally on all routes of transmission. It is more likely that a given intervention targets certain groups and routes of transmission, e.g. school closure. We found that our results for the all-transmission reduction carried over if only one transmission route was interrupted. While sustained school closure was found to have a major impact upon reducing the reproduction number of pandemic influenza 2009 in Hong Kong [38], many communities across the world found sustained school closure socially and economically costly [39]–[42]. Even in developed countries, the decision of when to reopen schools is challenging, and concurring with our results here, it has been argued that schools can be reopened only if population herd-immunity has been reached [14]. Earlier finding that an increase in the number of detected symptomatic cases needed to trigger a school closure reduced the total cumulative attack rates [39], [43] can be explained by our study. Increasing the population trigger for school closure (before it reaches its optimum) will lead to a smaller susceptible population that is closer to the critical threshold level when schools close, and thus will reduce the size of the ‘overshoot’ when the schools re-open. Therefore, as our results suggest, it may not be wise to close schools too early given the usual limited length of a school closure [14], [39], [43].

Our study comes with the usual caveats. We deliberately used a simple model to more thoroughly understand the relationship between the parameters and the outcomes. This somewhat limits its direct applicability, though as described here, certain features, such as population heterogeneity, might be less important for determining optimal interventions than expected. Still, for application to intervention planning, it might be useful to implement the ideas described here with a more detailed model, such as those described in ref. [15]–[24].

We also focused on a single strain and assumed complete immunity to re-infection. While this is likely applicable to influenza outbreaks in a single season, the model would need to be extended to include delayed or waning immunity [44], [45] to allow for reinfection by the same strain [46], or to include multiple strains to allow for features such as antigenic shift or the potential rise of drug-resistant strains [4]–[10], [12], [13].

We also acknowledge that the assumption of no natural birth (and therefore no increase in susceptible population) over the time course of one to three years may not necessarily hold in settings where birth rate is high.

We focused here on cumulative attack rates as the outcome and our goal was to minimize them. Other considerations, such as logistics and economics, may also factor into decisions for intervention choices. As shown in this study, if different interventions can lead to the same optimal state (i.e. achieve the same benefit), such additional considerations should be considered and modelled.

In summary, our study showed that even for relatively low inter-group transmission, at levels that were likely to occur for pathogens such as influenza, the population became synchronized enough to essentially consider them one homogenous population for control purposes. We found that it was best to choose an easily observable sub-population (e.g. schoolchildren) and measure their infection status in real time. Once the right number of infections had accrued (which was determined by the efficacy of the intervention and the length it could be applied), control measures should be started. The proper timing ensured that the control was optimal in the sense that it minimized the attack rate and the level of susceptible people dropped to the level at which herd immunity was reached, but not below.

## Supporting Information

Appendix S1 **The online Supporting Information file contains Appendix S1A ‘Total population trigger’ and Appendix S1B ‘The overlap of CA_AA_, CA_CC_ and CA_W_’, in addition to** **Figures 5** **,** **9** **and S1.** (Figures 5 and 9 are the same numbered figures as in the main text.)
(PDF)

## References

[1] Handel A, Longini IM, Antia R (2007). What is the best control strategy for multiple infectious disease outbreaks?. Proc Biol Sci.

[2] Anderson RM, May RM (1991). Infectious diseases of humans : dynamics and control.. Oxford; New York: Oxford University Press.

[3] Keeling MJ, Rohani P (2008). Modeling infectious diseases in humans and animals.. Princeton: Princeton University Press.

[4] Mills CE, Robins JM, Bergstrom CT, Lipsitch M (2006). Pandemic influenza: risk of multiple introductions and the need to prepare for them.. PLoS Med.

[5] Lipsitch M, Robins JM, Mills CE, Bergstrom CT (2006). Multiple outbreaks and flu containment plans.. Science.

[6] Lipsitch M, Cohen T, Murray M, Levin BR (2007). Antiviral resistance and the control of pandemic influenza.. PLoS Med.

[7] Wessel L, Hua Y, Wu J, Moghadas SM (2011). Public health interventions for epidemics: implications for multiple infection waves.. BMC Public Health.

[8] Handel A, Longini IM, Antia R (2009). Antiviral resistance and the control of pandemic influenza: the roles of stochasticity, evolution and model details.. J Theor Biol.

[9] Moghadas SM (2008). Management of drug resistance in the population: influenza as a case study.. Proc Biol Sci.

[10] Moghadas SM, Bowman CS, Rost G, Wu J (2008). Population-wide emergence of antiviral resistance during pandemic influenza.. PLoS One.

[11] Hansen E, Day T, Arino J, Wu J, Moghadas SM (2010). Strategies for the use of oseltamivir and zanamivir during pandemic outbreaks.. Can J Infect Dis Med Microbiol.

[12] Alexander ME, Bowman CS, Feng Z, Gardam M, Moghadas SM (2007). Emergence of drug resistance: implications for antiviral control of pandemic influenza.. Proc Biol Sci.

[13] Alexander ME, Dietrich SM, Hua Y, Moghadas SM (2009). A comparative evaluation of modelling strategies for the effect of treatment and host interactions on the spread of drug resistance.. J Theor Biol.

[14] Cauchemez S, Ferguson NM, Wachtel C, Tegnell A, Saour G (2009). Closure of schools during an influenza pandemic.. Lancet Infect Dis.

[15] Germann TC, Kadau K, Longini IM, Macken CA (2006). Mitigation strategies for pandemic influenza in the United States.. Proc Natl Acad Sci U S A.

[16] Longini IM, Nizam A, Xu S, Ungchusak K, Hanshaoworakul W (2005). Containing pandemic influenza at the source.. Science.

[17] Halloran ME, Ferguson NM, Eubank S, Longini IM, Cummings DA (2008). Modeling targeted layered containment of an influenza pandemic in the United States.. Proc Natl Acad Sci U S A.

[18] Hollingsworth TD, Ferguson NM, Anderson RM (2006). Will travel restrictions control the international spread of pandemic influenza?. Nat Med.

[19] Ferguson NM, Cummings DA, Cauchemez S, Fraser C, Riley S (2005). Strategies for containing an emerging influenza pandemic in Southeast Asia.. Nature.

[20] Ferguson NM, Cummings DA, Fraser C, Cajka JC, Cooley PC (2006). Strategies for mitigating an influenza pandemic.. Nature.

[21] Cauchemez S, Valleron AJ, Boelle PY, Flahault A, Ferguson NM (2008). Estimating the impact of school closure on influenza transmission from Sentinel data.. Nature.

[22] Ferguson NM, Mallett S, Jackson H, Roberts N, Ward P (2003). A population-dynamic model for evaluating the potential spread of drug-resistant influenza virus infections during community-based use of antivirals.. J Antimicrob Chemother.

[23] Wu JT, Leung GM, Lipsitch M, Cooper BS, Riley S (2009). Hedging against antiviral resistance during the next influenza pandemic using small stockpiles of an alternative chemotherapy.. PLoS Med.

[24] Coburn BJ, Wagner BG, Blower S (2009). Modeling influenza epidemics and pandemics: insights into the future of swine flu (H1N1).. BMC Med.

[25] White LF, Wallinga J, Finelli L, Reed C, Riley S (2009). Estimation of the reproductive number and the serial interval in early phase of the 2009 influenza A/H1N1 pandemic in the USA.. Influenza Other Respi Viruses.

[26] Fraser C, Donnelly CA, Cauchemez S, Hanage WP, Van Kerkhove MD (2009). Pandemic potential of a strain of influenza A (H1N1): early findings.. Science.

[27] Nishiura H, Castillo-Chavez C, Safan M, Chowell G (2009). Transmission potential of the new influenza A(H1N1) virus and its age-specificity in Japan.. Euro Surveill 14: pii.

[28] Boëlle PY, Bernillon P, Desenclos JC (2009). A preliminary estimation of the reproduction ratio for new influenza A(H1N1) from the outbreak in Mexico, March-April 2009.. Euro Surveill 14: pii.

[29] Hahne S, Donker T, Meijer A, Timen A, van Steenbergen J (2009). Epidemiology and control of influenza A(H1N1)v in the Netherlands: the first 115 cases.. Euro Surveill 14: pii.

[30] Yang Y, Sugimoto JD, Halloran ME, Basta NE, Chao DL (2009). The transmissibility and control of pandemic influenza A (H1N1) virus.. Science.

[31] Pourbohloul B, Ahued A, Davoudi B, Meza R, Meyers LA (2009). Initial human transmission dynamics of the pandemic (H1N1) 2009 virus in North America.. Influenza Other Respi Viruses.

[32] Nishiura H, Wilson N, Baker MG (2009). Estimating the reproduction number of the novel influenza A virus (H1N1) in a Southern Hemisphere setting: preliminary estimate in New Zealand.. N Z Med J.

[33] Munayco CV, Gomez J, Laguna-Torres VA, Arrasco J, Kochel TJ (2009). Epidemiological and transmissibility analysis of influenza A(H1N1)v in a southern hemisphere setting: Peru.. Euro Surveill 14: pii.

[34] Read JM, Eames KT, Edmunds WJ (2008). Dynamic social networks and the implications for the spread of infectious disease.. J R Soc Interface.

[35] Keeling MJ, Eames KT (2005). Networks and epidemic models.. J R Soc Interface.

[36] Horby P, Pham QT, Hens N, Nguyen TT, Le QM (2011). Social contact patterns in Vietnam and implications for the control of infectious diseases.. PLoS One.

[37] Kermack WO, McKendrick AG (1927). A Contribution to the Mathematical Theory of Epidemics.. Proceedings of the Royal Society of London Series A.

[38] Wu JT, Cowling BJ, Lau EH, Ip DK, Ho LM (2010). School closure and mitigation of pandemic (H1N1) 2009, Hong Kong.. Emerg Infect Dis.

[39] Brown ST, Tai JH, Bailey RR, Cooley PC, Wheaton WD (2011). Would school closure for the 2009 H1N1 influenza epidemic have been worth the cost?: a computational simulation of Pennsylvania.. BMC Public Health.

[40] House T, Baguelin M, Van Hoek AJ, White PJ, Sadique Z (2011). Modelling the impact of local reactive school closures on critical care provision during an influenza pandemic.. Proc Biol Sci.

[41] House T, Baguelin M, van Hoek AJ, Flasche S, White P (2009). Can reactive school closures help critical care provision during the current influenza pandemic?. PLoS Curr.

[42] Sadique MZ, Adams EJ, Edmunds WJ (2008). Estimating the costs of school closure for mitigating an influenza pandemic.. BMC Public Health.

[43] Lee BY, Brown ST, Cooley P, Potter MA, Wheaton WD (2010). Simulating school closure strategies to mitigate an influenza epidemic.. J Public Health Manag Pract.

[44] Camacho A, Ballesteros S, Graham AL, Carrat F, Ratmann O (2011). Explaining rapid reinfections in multiple-wave influenza outbreaks: Tristan da Cunha 1971 epidemic as a case study.. Proc Biol Sci.

[45] Mathews JD, McCaw CT, McVernon J, McBryde ES, McCaw JM (2007). A biological model for influenza transmission: pandemic planning implications of asymptomatic infection and immunity.. PLoS One.

[46] Perez CM, Ferres M, Labarca JA (2010). Pandemic (H1N1) 2009 reinfection, Chile.. Emerg Infect Dis.

[47] Carrat F, Vergu E, Ferguson NM, Lemaitre M, Cauchemez S (2008). Time lines of infection and disease in human influenza: a review of volunteer challenge studies.. Am J Epidemiol.

[48] Frank AL, Taber LH, Wells CR, Wells JM, Glezen WP (1981). Patterns of shedding of myxoviruses and paramyxoviruses in children.. J Infect Dis.

