# Supplementary material for: How to Minimize the Attack Rate during Multiple Influenza Outbreaks in a Heterogeneous Population
Source: PLoS One. 2012 Jun 11;7(6):e36573. doi: 10.1371/journal.pone.0036573 (PMC3372524; doi:10.1371/journal.pone.0036573)
Supplement: Appendix S1 — The online Supporting Information file contains Appendix S1A ‘Total population trigger’ and Appendix S1B ‘The overlap of CAAA, CACC and CAW’, in addition to Figures 5 , 9 and S1. (Figures 5 and 9 are the same numbered figures as in the main text.) (PDF) [file pone.0036573.s001.pdf]

How to minimize the attack rate during multiple influenza outbreaks in a heterogeneous population

PLoS ONE: 10.1371/journal.pone.0036573

Isaac Chun-Hai Fung, Rustom Antia, Andreas Handel

Appendix S1A: Total population trigger

While in the main text we used the cumulative proportion of children infected to trigger an intervention, here we considered a scenario in which the intervention was triggered when the cumulative proportion of *total infected population* reached a certain threshold (Figure S1). Compared to the case using the children's population trigger (Figure 5, from main article), when using the total population trigger, two minima were observed in the absence of coupling ($\beta_{AC} = \beta_{CA} = 0$) in the CA_W curve with the exception of the 10% scenario where the adults' minimum was too small to have any impact on CA_W (Figure S1). In the presence of coupling, the results were similar between the children's trigger (Figure 9, from main article) and that of the total population (Figure S1). In both cases, two CA_W minima were observed when coupling level was very small ($\beta_{AC} = 0.01 * \beta_{AA}$; $\beta_{CA} = 0.01 * \beta_{CC}$) for 30%, 50% and 70% adults, and the CA_{AA}, CA_{CC} and CA_W overlapped when $\beta_{AC} = \beta_{AA}$ and $\beta_{CA} = \beta_{CC}$ (Figures 9, from main article and S1). As we would expect, if one population sub-group predominated in the population, the impact of the smaller group on the CA_w was minimal regardless of the choice of trigger. Again, similar to the children's population trigger, for each population composition, there was an infected population trigger level above which the intervention could not be triggered as it was the maximum total cumulative attack rate attained by the epidemic (after the re-introduction of the infection) in the absence of any intervention (Figure S1).

Figure S1 Cumulative attack rates against the total population trigger (defined as cumulative proportion of *total population* infected). $\beta_{AC} = \beta_{CA} = 0$ (upper row); $\beta_{AC} = 0.01 * \beta_{AA}$, $\beta_{CA} = 0.01 * \beta_{CC}$ (second upper row); $\beta_{AC} = 0.1 * \beta_{AA}$, $\beta_{CA} = 0.1 * \beta_{CC}$ (second lower row); $\beta_{AC} = \beta_{AA}$, $\beta_{CA} = \beta_{CC}$ (lower row). Black dotted line: adults (CA_{AA}); grey broken line: children (CA_{CC}); red solid line: total population (CA_W). The x-axis does not go beyond a threshold level of 0.8, since in none of these scenarios the CA_W reached a higher attack rate among the total population and therefore intervention would not be triggered. Proportion of adults in population (left to right): 10%, 30%, 50%, 70%, 90%. $R_{0A} = 1.25$, $R_{0C} = 2$; long intervention; interrupt all routes of transmission; intervention efficacy, $f_{AA} = f_{AC} = f_{CA} = f_{CC} = 1$. All other parameters and initial conditions are listed in Tables 1 and 2.

Appendix S1 B: The overlap of CA_{AA}, CA_{CC} and CA_W

In the following, we derived an analytic explanation for the feature that the CA_{AA}, CA_{CC} and CA_W overlapped with each other when $\beta_{AC} = \beta_{AA}$ and $\beta_{CA} = \beta_{CC}$, as observed in the lower panels of Figure 8 and discussed in the Results Section, under sub-section "Coupling through inter-group transmission".

If we let $\beta_1 = \beta_{AC} = \beta_{AA}$; $\beta_2 = \beta_{CA} = \beta_{CC}$; $f = f_{AA} = f_{AC} = f_{CA} = f_{CC}$,

then from equations (1) and (2),

$$\dot{S}_A = -(1-f)\beta_1 S_A I_A - (1-f)\,\beta_2 S_A I_C$$

$$\dot{S}_C = -(1-f)\,\beta_2 S_C I_C - (1-f)\beta_1 S_C I_A$$

Since the force of infection, $\lambda = (1-f)\,(\beta_1 I_A + \beta_2 I_C)$, therefore,

$$\dot{S}_A / \dot{S}_C = (-\lambda S_A) / (-\lambda S_C) = S_A / S_C$$

As $\dot{S}_A \approx \lim_{\Delta t \to 0} (S_{A0} - S_A)/t$ and $\dot{S}_C \approx \lim_{\Delta t \to 0} (S_{C0} - S_C)/t$,

Therefore,

$$(S_{A0} - S_A) / (S_{C0} - S_C) = S_A / S_C$$

$$S_{A0} / S_A = S_{C0} / S_C$$

$$1 - (S_A / S_{A0}) = 1 - (S_C / S_{C0})$$

From the calculations above, we can tell that $CA_{AA} = CA_{CC} = CA_W$ as long as these two conditions are satisfied:

(a) $\beta_{AC} = \beta_{AA}$ and $\beta_{CA} = \beta_{CC}$; and
(b) $f_{AA} = f_{AC} = f_{CA} = f_{CC}$.

This holds true regardless of the exact values of R_{0A} and R_{0C}, and regardless of how the reproduction numbers related to the transmission coefficients.

Figure 5

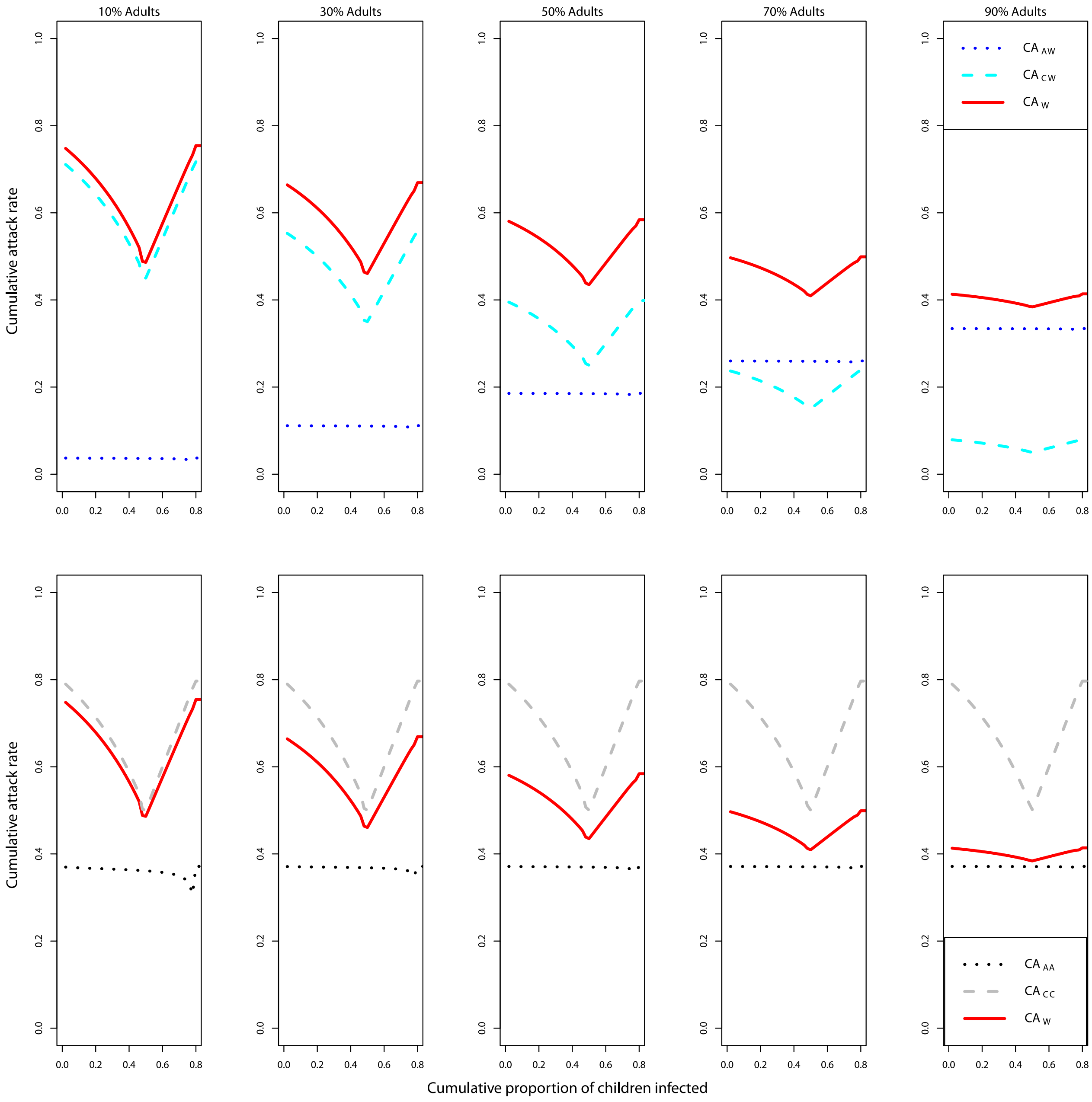

10% Adults
30% Adults
50% Adults
70% Adults
90% Adults
Cumulative attack rate
CA AW
CA CW
CA W
CA AA
CA CC
CA W
Cumulative proportion of children infected

Figure 9

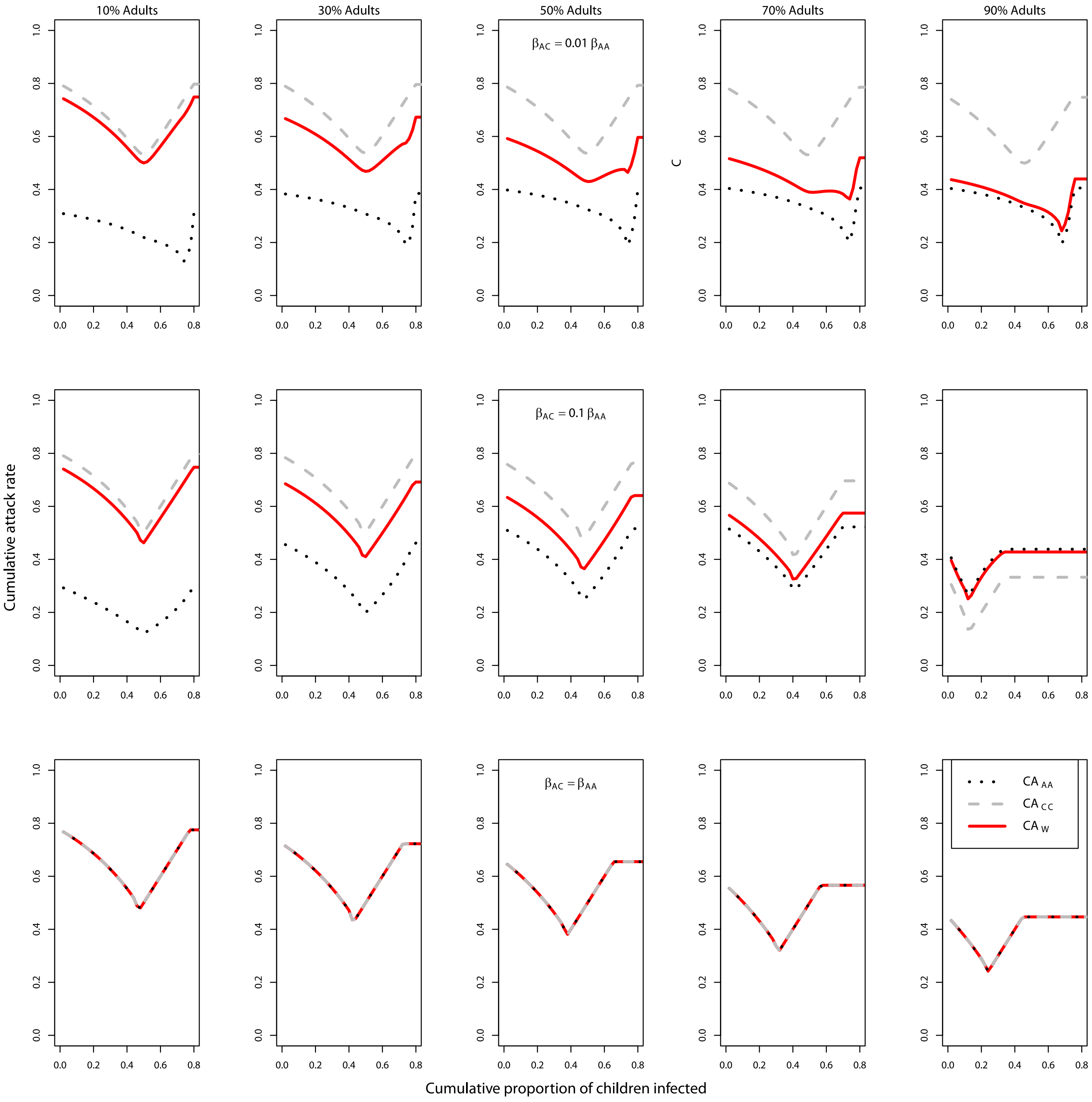
10% Adults
30% Adults
50% Adults
70% Adults
90% Adults
$\beta_{AC} = 0.01\,\beta_{AA}$
$\beta_{AC} = 0.1\,\beta_{AA}$
$\beta_{AC} = \beta_{AA}$
C
Cumulative attack rate
Cumulative proportion of children infected
CA_{AA}
CA_{CC}
CA_{W}

Figure S1

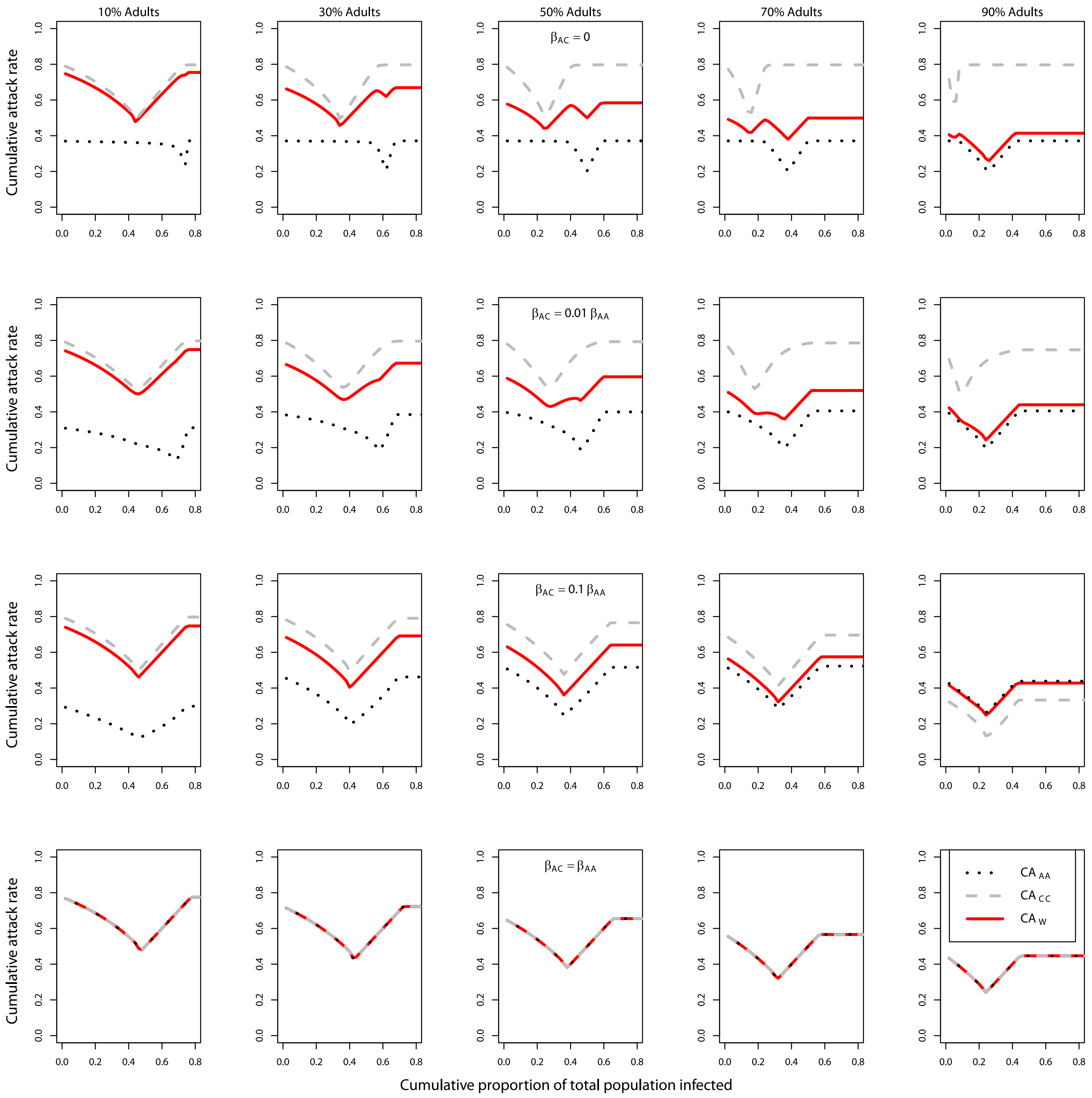
10% Adults
30% Adults
50% Adults
70% Adults
90% Adults
$\beta_{AC} = 0$
$\beta_{AC} = 0.01\ \beta_{AA}$
$\beta_{AC} = 0.1\ \beta_{AA}$
$\beta_{AC} = \beta_{AA}$
Cumulative attack rate
CA_{AA}
CA_{CC}
CA_{W}
Cumulative proportion of total population infected
